# Variability of Grip Kinetics during Adult Signature Writing

**DOI:** 10.1371/journal.pone.0063216

**Published:** 2013-05-02

**Authors:** Bassma Ghali, Nayanashri Thalanki Anantha, Jennifer Chan, Tom Chau

**Affiliations:** 1 Bloorview Research Institute, Holland Bloorview Kids Rehabilitation Hospital, Toronto, Ontario, Canada; 2 Institute of Biomaterials and Biomedical Engineering, University of Toronto, Toronto, Ontario, Canada; 3 Engineering Science, University of Toronto, Toronto, Ontario, Canada; McMaster University, Canada

## Abstract

Grip kinetics and their variation are emerging as important considerations in the clinical assessment of handwriting pathologies, fine motor rehabilitation, biometrics, forensics and ergonomic pen design. This study evaluated the intra- and inter-participant variability of grip shape kinetics in adults during signature writing. Twenty (20) adult participants wrote on a digitizing tablet using an instrumented pen that measured the forces exerted on its barrel. Signature samples were collected over 10 days, 3 times a day, to capture temporal variations in grip shape kinetics. A kinetic topography (i.e., grip shape image) was derived per signature by time-averaging the measured force at each of 32 locations around the pen barrel. The normalized cross correlations (NCC) of grip shape images were calculated within- and between-participants. Several classification algorithms were implemented to gauge the error rate of participant discrimination based on grip shape kinetics. Four different grip shapes emerged and several participants made grip adjustments (change in grip shape or grip height) or rotated the pen during writing. Nonetheless, intra-participant variation in grip kinetics was generally much smaller than inter-participant force variations. Using the entire grip shape images as a 32-dimensional input feature vector, a K-nearest neighbor classifier achieved an error rate of 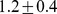
% in discriminating among participants. These results indicate that writers had unique grip shape kinetics that were repeatable over time but distinct from those of other participants. The topographic analysis of grip kinetics may inform the development of personalized interventions or customizable grips in clinical and industrial applications, respectively.

## Introduction

Handwriting grip is the arrangement of the fingers and thumb around the barrel of a writing instrument for the production of written output. Recent advances in instrumented writing utensils [1]–[3] have enabled the measurement of handwriting grip kinetics, i.e., the forces exerted by the fingers and thumb on the barrel of the writing implement during handwriting. Handwriting grip kinetics are emerging as an important quantitative measure in the clinical domain, but may also have relevance in biometrics, forensics and ergonomics.

### Clinical Assessments

Recent clinical handwriting studies have expanded from pen tip kinematics to pen-hand contact kinetics [4]–[8]. In a sample of patients with writer’s cramp (WC), Schneider et al. [9] discovered significant elevation of grip forces above the levels of healthy participants only for those with dystonic WC, and thus suggested that grip kinetics may uniquely provide clinical subtype differentiation. Likewise, Hermsdörfer et al. [8] reported that exaggerated forces in patients with WC occurred more frequently than abnormal kinematics, concluding that grip force is an important descriptor of individual impairment characteristics that are independent of writing kinematics. This finding corroborates earlier conclusions by Fernandes and Chau [10] that the dynamics of grip force and pacing are independently regulated.

### Rehabilitation

In addition to the clinical characterization of handwriting function, grip force has a role in both treatment and outcome measurement. Baur et al. [3] developed a novel intervention for patients with writer’s cramp, using auditory grip force feedback, namely, a continuous low frequency tone whose pitch increased with escalating grip force. Significant reduction in writing pressures and pain were noted over 7 sessions of treatment. Deploying grip force as an outcome measure, Baur et al. [11] found that both a modified pen grip and handwriting training (motor exercises) decreased grip force in patients with writer’s cramp and in a sample of asymptomatic controls.

### Biometrics

Online and offline writer identification and signature verification studies have investigated the intra- and inter-participant variability of handwriting [12]–[14]. However, these studies have focused exclusively on normal forces, and kinematic (e.g., position, velocity, acceleration, inclination angle), spatiotemporal (e.g., stroke durations, stroke length, in-air time) and image-based features. The biometric value of grip patterns and its associated kinetics has yet to be explored in handwriting studies. In gun control applications, for example, grip force patterns have already proven valuable for biometric verification [15]–[17].

### Forensics

Given the relationship between axial (pen tip force on the writing surface along the length of the pen) and grip forces, knowledge of the former from an analysis of paper indentations [18] may shed light on the pen grip of the writer and possibly the presence of musculoskeletal pathologies of the writer.

### Ergonomics

Grip forces may inform the design of new pens, such as in [19], which proposed a pen with a flared silicon grip area as a means of reducing muscle load (EMG activation) and upper limb pain during extended periods of continuous writing.

Given the emerging importance of grip kinetics, in this study we systematically evaluated the intra- and inter-participant variability of grip shape and forces in an adult population during signature writing.

## Methods

### Ethics Statement

The protocol of the study was approved by the research ethics boards of Holland Bloorview Kids Rehabilitation Hospital and the University of Toronto. Each participant provided an informed written consent.

### Participants

We recruited a convenient sample of 20 adult participants (8 males; 17 right-handed; age 27±6 years) with no history of musculoskeletal injuries or neurological impairments. Each participant completed a simple demographic questionnaire upon acceptance to participate in the study. The questionnaire asked about gender, handedness, age, occupation, education level, fathers education level, mothers education level and racial/ethnic group.

### Instrumentation

**Figure 1:**
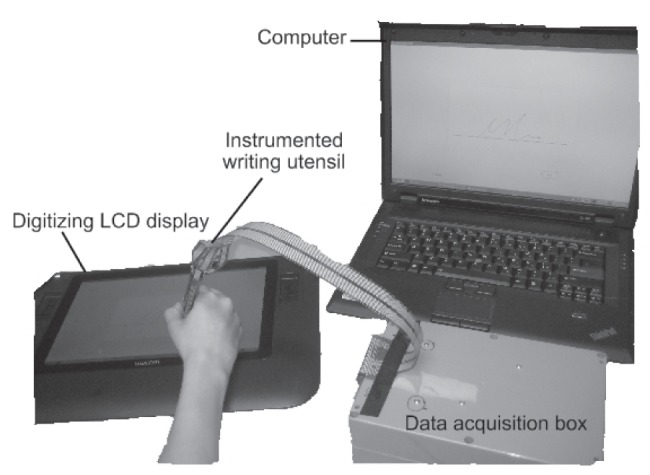
Data Collection instrumentation setup. Participants wrote with the instrumented writing utensil on a digitizing LCD display. The data acquisition box and the computer transmitted and saved the data respectively.

Figure 1 depicts the study equipment, which consisted of an instrumented writing utensil and a digitizing LCD display. The utensil was constructed by inserting the electronics of a Wacom 6D Art Pen inside a cylindrical barrel machined out of Delrin. To capture grip forces, an array of 64 Tekscan 9811 force sensors were first adhered to the pen barrel using an adhesive (3 M Super 77 Multipurpose Spray Adhesive) and then taped down to mitigate sensor peeling. Note that in sensor calibration and data collection, only the 32 sensors closest to the apex of the pen were considered as the more distal sensors were generally not activated during writing. Four such pens were manufactured for this study. See [1] for further details about utensil construction. The writing utensil was connected to a data collection computer via a custom-made data acquisition box containing operational amplifier circuits that biased voltages to maximize the input signal resolution, multiplexers and a 16 analog input data acquisition card. Grip forces were sampled at 250 Hz. The force sensor array was replaced multiple times during data collection due to sensor wear and tear. The total pen weight was 24 g. The pen had a diameter of 1.3 cm and a height of 14 cm. The writing surface was an electronically inking Wacom Cintiq 12WX digitizing LCD display, which collected axial force, pen tip position and pen angles (rotation, altitude and azimuth) at a frequency of 105 Hz. The digitizing display was connected to the data collection computer via VGA and USB cables.

### Calibration Set-up and Procedure

**Figure 2:**
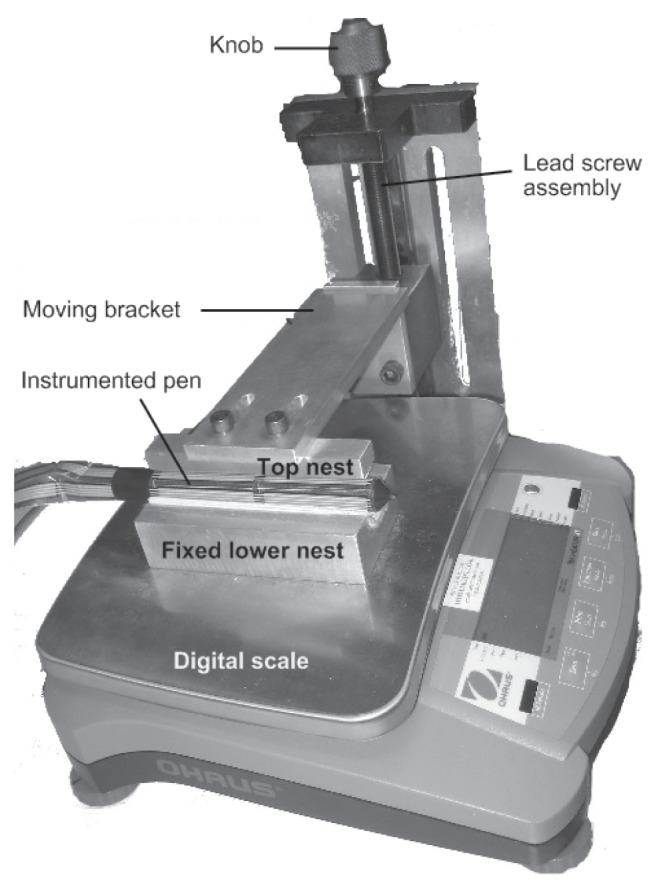
The calibration setup. Each sensor in the force sensor array was calibrated using this setup through loading and unloading of the sensors.

Prior to data collection, the force sensors on the barrel of the writing utensils were systematically calibrated. Figure 2 portrays the calibration setup, which included a digital scale that measured the applied load, a fixed lower nest on which the pen rested, a top nest that loaded the pen from above, and, a lead screw assembly that raised and lowered the top nest via a rotary knob and moving bracket. To accelerate calibration, the top nest was designed to simultaneously load a column of eight sensors at one time. Specifically, the top nest was contoured to match the curvature of the pen’s barrel and padded with a thin layer of vinyl (CON-TACT non-adhesive liner) to encourage uniform distribution of force along the targeted section of the pen barrel. To avoid slippage of the pen, the fixed bottom nest was similarly padded.

The pen was placed on the bottom nest with the targeted column of sensors facing up. The column of sensors was gradually loaded and unloaded by rotating the knob. The load on an individual sensor ranged from 0 to 1100 grams. Force and digital scale readings were synchronized and recorded directly to a computer via the custom-made data acquisition box and a serial cable, respectively. From these data, loading and unloading curves were derived offline. These calibration curves facilitated the translation of subsequent sensor readings (in Volts) into physical units of force (Newtons).

Each time a pen was calibrated, the above loading and unloading procedure was repeated 6 times, 3 with the pen tip pointing in one direction and 3 with the pen in the opposite direction. Averaging calibration curves from these iterations helped to minimize the effect of any differences due to misalignment between the top nest and sensor bank, and any orientation-dependent load imbalance.

Throughout the data collection described below, the force sensors for each pen were calibrated every 2 to 3 days to account for possible changes in sensor behavior over time, especially a decrease in sensor sensitivity with usage.

### Data Collection Protocol

All data collection took place in a laboratory within a university teaching hospital. Each session adhered to the following steps:

1. Participants sat comfortably on a height-adjustable task chair, facing a typical workbench. Participants wore a grounding strap on the non-dominant hand and held the instrumented utensil with the dominant hand.
2. The force sensors were checked by a researcher through visual inspection of a real-time colour display of individual sensor force values.
3. A custom software ‘wizard’ was launched and systematically guided participants through each step of data collection.
4. The participant answered a status question on the tablet, namely, “Do you think there is any emotional, mental or biomechanical factors that can affect your handwriting now? (E.g. angry, stressed, nervous, sick, muscle stiffness or fatigue). The answer should be yes or no.”
5. The participant was asked to hold the distal end of the pen without contacting any of the sensors on the pen for a 10 second period to collect baseline values of the 32 force sensors on the pen. These baseline values are used to calculate the pre-grip values of each force sensor as explained in taring and calibration procedures below.
6. The participant held the pen naturally and provided 20 samples of a well-practiced bogus signature that each participant practiced for two weeks prior to data collection. The participant signed on the tablet within a delineated area, which was refreshed by an explicit button press after each signature.
7. Two digital photos, one a dorsal view and the other a palmar view of the hand grip, were taken at the halfway point of the session.
8. The participant provided 20 samples of his/her own authentic signature.
9. Two digital photos (dorsal and palmar views) of the hand grip were taken at the end of each session.

The above procedure was repeated three times a day (morning, afternoon, and evening), on 10 different days according to participant availability. On average, data collection was completed in 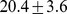 days. The iterative collection was designed to capture grip shape and kinetic variations over time. For each participant, 600 authentic signatures and 600 well-practiced bogus signatures were obtained over a total of 30 sessions (3 sessions per day×10 days). In this paper, we only consider the 12000 (600 signatures×20 participants) authentic signatures. A researcher noted any writing mistakes during data collection or any suspicious sensors during calibration, to inform subsequent data screening.

### Data Preprocessing

#### Clean-up

Upon visual review of the collected data and cross-referencing with researcher notes, we discarded 225 authentic signatures out of the 12,000 for one or more of the following reasons: visible mistakes while writing the signature (e.g., scratched out text), an extended pause in the midst of a signature, or an obvious force sensor malfunction (e.g., loss of signal). Also, signature samples accidentally contaminated with extra lines or dots on the tablet before or after the signature were salvaged by trimming the contaminant data from the beginning or end of the signature sample as appropriate.

The force data were subjected to a sixth order Butterworth low-pass filter with a cutoff frequency of 10 Hz, which was deemed to be the lowest frequency below which more than 95% of the signal power resided. Signatures that exhibited visible low frequency oscillations unrelated to handwriting (likely noise from nearby electronic devices) were excluded from the subsequent analysis. The total number of samples that were excluded at this stage was 735 authentic signatures. In total, 8% of the 12,000 authentic signatures were excluded subsequent to data cleanup, leaving 11,040 signatures for analysis, with an average of 552 samples per participant.

#### Taring and calibration

Since the force sensors were curved around the barrel of the pen, they had non-zero readouts prior to the participant gripping the pen. These pre-grip values were estimated using the 10 second baseline collected prior to any handwriting, on a per sensor, per session basis. For each sensor, the mean pre-grip value was subtracted from all subsequent grip force data in a given session. In this way, the readout of each sensor prior to the participant gripping the pen was zero. Each sensor reading was then translated into units of physical force (N) via a least-squares second order polynomial fit to the corresponding shifted calibration data, as shown below:
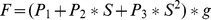
(1)where 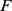 is the calibrated sensor reading in Newtons, 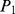, 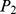 and 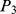 are the polynomial coefficients, 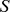 is the raw reading for a particular sensor, and 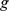 is the gravitational constant (9.81 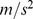).

**Figure 3:**
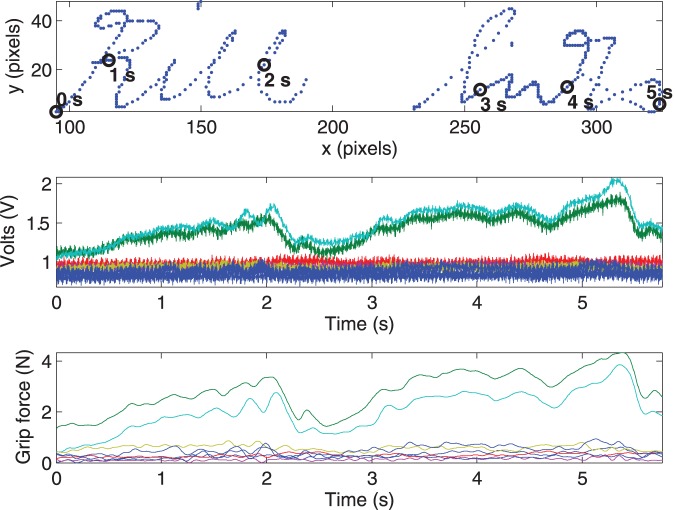
A signature example and the associated grip force signals. The top graph shows the position signals of a signature sample annotated at 1 second increments, and the middle and bottom graphs show the associated raw and processed grip force signals respectively. For clarity, only the non-zero force traces are shown in the latter. In bottom two graphs, each line represents the readout of a different grip sensor. Note that this sample is not an authentic signature; it is a sample of a well-practiced signature.

Figure 3 shows one signature sample and the associated raw and processed (trimmed, filtered, shifted and calibrated) grip forces. Note that the signature shown in Figure 3 is a sample of the well-practiced rather than authentic signature. This sample was used to illustrate the relation of the writing sample to the raw and processed grip force data.

To provide the reader with a sense of the spatial and temporal characteristics of the authentic signatures considered in this paper, a list of key spatiotemporal features is presented in the results section below. The time and pen tip position (x and y coordinates) data collected by the LCD digitizing display were used to calculate duration, total path length, height, width and average speed of each signature. Note that these data were collected only when the writing instrument touched the digitizing display, which thus provided the onset and offset of writing. The height of each signature was calculated as the difference between the minimum and maximum position in the vertical direction. The width was calculated similarly but in the horizontal direction. The manufacturer-specified resolution of the digitizing display was used to convert the derived distances from pixels to millimeters. No other preprocessing was applied to these data.

### Data Analysis

#### Grip shape identification

By reviewing the collected photos, the grip shape of each participant was classified according to standard grip shape taxonomies [20]–[23]. To establish inter-rater reliability, a random sample of 20% of the photographs were examined by an independent occupational therapist not associated with the study. Complete (100%) agreement was achieved.

#### Deriving grip shape

**Figure 4:**
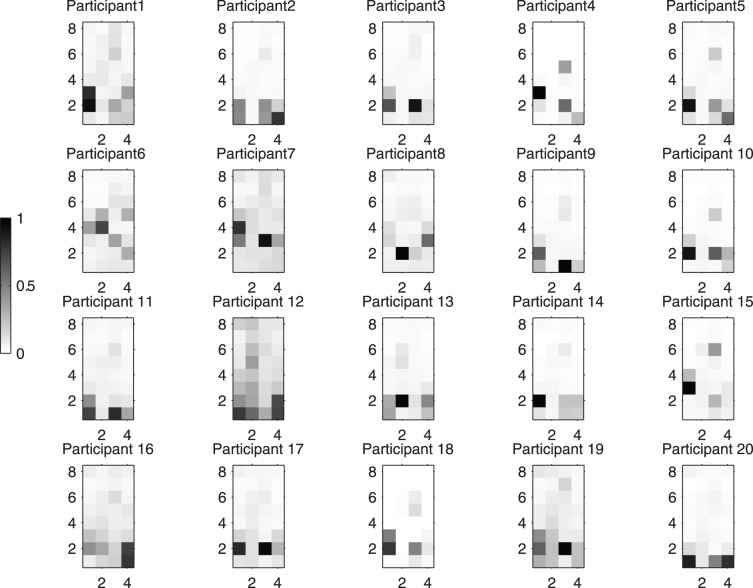
Mean grip shape images of the 20 participants. Each grip shape image represents the grip force distribution on the 4 by 8 force sensor array with the black points being the ones with the highest force.

The time-average of forces applied to each sensor over the course of a signature was computed. These average forces were arranged into a matrix corresponding to the spatial arrangement of sensors around the barrel. The resultant matrix was termed the grip shape, given that a heat map of this matrix (i.e., grip shape image) reveals the spatial distribution of forces around the barrel. Each signature thus had an associated grip shape matrix (See Figure 4 for an example). Note that for a given grip shape matrix, the forces were normalized to fall within 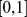. The distance, 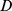, between two grip shape matrices, 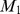 and 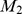, was computed as the Frobenius norm of their difference [24], i.e.,
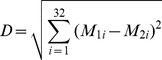
(2)where 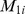 and 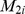 are the 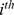 entries of the respective grip shape matrices. For each signature’s grip shape matrix, we further computed the mean of the distances to the grip shape matrices of all other signatures by the same participant, and termed this the *discrepancy* measure for that signature. Grip shape matrices with discrepancy measure falling within the lowest 50% were then averaged across signatures to arrive at the mean grip shape for each participant. In short, the mean grip shape for each participant was an average of the signature-specific force distributions across signatures with the most typical grip shape for that participant.

#### Grip shape variation

The intra- and inter-participant variability of the grip shape was studied in three different ways.

1. Fisher’s ratio of 2-dimensional normalized cross-correlation (NCC) between two grip shape images: Intra-participant differences were estimated by the NCC between a participant’s mean grip shape and the grip shape images of all other signatures of the same participant. Inter-participant differences were captured by the NCC between the participant’s mean grip shape and the grip shape images of signatures of all other participants. Fisher’s ratio [25] was used to quantify the separation between distributions of intra- and inter-participant NCC values, namely,
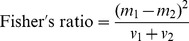
(3)where 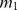 and 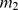 are the means, and 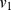 and 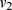 are the variances of the two distributions.
2. Error rates of discriminating NCC values among writers: Three types of classifiers, namely, linear discriminant analysis (LDA), K-nearest neighbor (KNN) and back-propagation neural networks (NN), were invoked. For KNN, we considered K-values of 1,3 and 5 while the NN had an architecture of 1-10-1 (input-hidden-output units). In each case, one classifier was trained per participant to determine if a signature belonged to that participant or not. For a given writing sample, the input to the 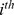 classifier (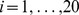) was a one-dimensional NCC value between the unknown signature and mean grip shape of the 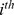 participant. The one dimensional binary output denoted the predicted membership of the unknown signature (1 = belongs to 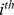 participant). For each participant-specific classifier, the training set included intra-participant NCC values with a desired output of 1 and an equal number of inter-participant NCC values with a desired output of zero. Inter-participant NCC values were pseudo-randomly selected to ensure representation from all participants. The misclassification rate was calculated for all these classifiers using 10-fold cross validation.
3. Error rate of discriminating grip shape matrices among writers: We discriminated among writers using the entire 32-element grip shape matrix as the input to multiclass LDA and KNN classifiers with a single output denoting the participant number. A backpropagation multiclass NN classifier with an architecture of 32-25-5 was also tested. In this case, the 5 digit output was a binary representation of the participant number. Misclassification rate was estimated using 10-fold cross validation for all these classifiers.

Pen rotation within- and between-sessions may have led to spatial misalignment of grip shape images. To mitigate these rotational effects, the grip shape images of each participant were aligned horizontally to the mean grip shape image of the same participant using the horizontal offsets that maximized the NCC between the mean and individual grip shape images. Here, horizontal refers to the circumferential axis. We then repeated the grip shape variation analyses described above post-alignment.

## Results

### Spatiotemporal Features

Table 1 summarizes the duration, total path length, height, width and average speed of the authentic signatures across participants. Clearly, signatures ranged in duration, size and speed. The duration of the signatures ranged from 1–6 seconds, depending on the writing speed of the particular participant and the length of the individual’s signature. An earlier study that collected signatures from 70 participants reported that the duration ranged from 2–10 seconds [26]. According to the size taxonomy in [1], the size of the signatures collected herein ranged from small to large. The values of average speed echo those reported in [28], which were based on 55 writers writing their authentic signature 30 times.

**Table 1:** Temporal, spatial and speed information of the authentic signatures.

	Duration (sec)		Total path length (mm)		Height (mm)		Width (mm)		Average speed (mm/sec)	
Participant	Mean	SD	Mean	SD	Mean	SD	Mean	SD	mean	SD
1	1.1	0.2	95	12	18	3	49	8	115	17
2	6.1	0.3	491	56	27	3	100	15	87	11
3	3.5	0.4	239	30	17	4	60	15	84	19
4	3.1	0.2	251	33	17	2	60	9	90	9
5	2.6	0.2	275	38	24	3	51	7	112	10
6	5.8	0.2	468	49	18	2	71	9	79	9
7	1.3	0.2	226	28	29	3	87	6	226	27
8	3.9	0.3	314	25	21	2	82	5	88	8
9	4.3	0.3	172	21	14	3	38	3	45	6
10	3.9	0.3	163	13	14	1	47	4	43	3
11	3.6	0.3	296	30	25	3	54	5	95	9
12	4.3	0.4	246	37	25	5	68	8	63	9
13	1.2	0.1	122	14	20	2	16	2	106	9
14	2.9	0.6	140	28	21	3	29	5	55	8
15	3.7	0.3	173	16	13	2	40	4	49	4
16	2.2	0.3	244	66	32	7	35	6	110	20
17	2.3	0.3	87	9	12	1	26	3	38	5
18	1.3	0.2	139	28	17	3	35	9	111	16
19	4.2	0.3	188	19	15	2	47	6	53	4
20	3.3	0.3	305	40	18	2	73	7	104	12
**Average**	**3.2**	**0.3**	**231.7**	**29.6**	**19.8**	**2.9**	**53.3**	**6.7**	**87.7**	**10.7**

SD (standard deviation).

### Grip Shapes

Table 2 lists the grip shape for each participant as determined via photo-review. Based on the observed grip shape variation, the participants were categorized into four groups, namely, (1) six who maintained a consistent grip shape throughout, (2) seven who rotated the pen and/or altered grip height either within or between sessions, (3) four who routinely changed grip shapes within and/or between sessions, and, (4) three who altered their grip shape after the initial sessions.

**Table 2:** Grip shape of each participant and associated observations.

Participant	Grip shape	Observations
1	Quadrupod	Rotated pen in some sessions
2	Dynamic tripod	Changed grip height and rotated pen slightly between sessions
3	Dynamic tripod	Rotated pen in most sessions
4	Lateral tripod	Occasionally started with a dynamic tripod grasp
5	Dynamic tripod	Rotated pen in some sessions
6	Quadrupod/other	Changed grip shape and grip height in most sessions
7	Static tripod	Consistent grip shape
8	Lateral tripod	Consistently used quadrupod grasp for the first 3 sessions but varied grip shape in other sessions
9	Dynamic tripod	Consistent grip shape
10	Dynamic tripod	Consistent grip shape
11	Quadrupod	Changed grip shape to lateral quadrupod grasp and rotated pen in some sessions
12	Quadrupod (left)	Rotated pen slightly
13	Static tripod (Left)	Consistent grip shape
14	Dynamic tripod	Changed grip shape in some sessions
15	Lateral tripod	Changed grip height between sessions
16	Quadrupod (left)	Changed grip after first session
17	Dynamic tripod	Rotated pen slightly
18	Dynamic tripod	Used different grip shape (quadrupod) for the first three sessions
19	Dynamic tripod	Consistent grip shape
20	Quadrupod	Consistent grip shape

### Topographical Analysis of Grip Shape Variability

Figure 4 shows the mean grip shape for each of the 20 participants. The topographic images represent the force distributions over the 8×4 force sensor array, with the bottom row of sensors being closest to the tip of the pen. The mean grip shape images appear to be unique among participants even when participants were categorized by photo-review as having the same, consistently employed grip shape (e.g., Participants 9, 10 and 19 all have dynamic tripod grasps).

Notice that there are generally a 2 to 4 focal areas of peak force and a blurring of lower forces elsewhere. Also note that most the force is concentrated near the apex of the pen and that the forces are distributed horizontally, presumably to provide stability to the utensil.

**Figure 5:**
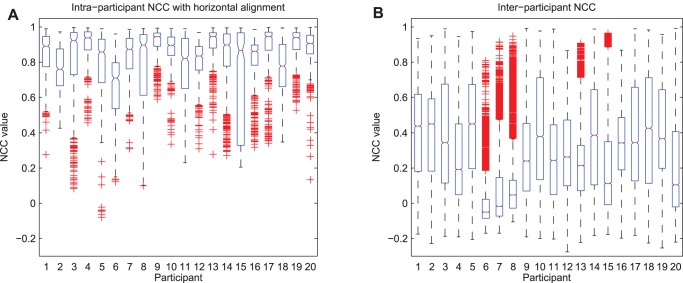
Box plots of the intra- (left panel A) and inter-participant (right panel B) NCC values for all 20 participants. Each box represents the distribution of NCC values for one participant, while ‘+’ symbols denote outliers (values beyond 1.5 interquartile ranges from the median).

Box plots of the intra- and inter-participant NCC for all 20 participants are shown in Figure 5. The plot on the left portrays the level of grip shape consistency within each participant based on the distribution of grip forces. Note that median NCC values are close to 1 and adorned with small boxes, suggesting high consistency of static grip shape images for a given participant, over all 30 sessions. It is worthy to note that some participants (4, 9, 10, 13, 17, 19, and 20) were more consistent than others (3, 6, 11, 15, and 18). In most cases, this finding resonates with the observations made in Table 2 which is a descriptive characterization of the grip shape based on retrospective photo review only (i.e., that some participants were consistent whereas others altered their grip shapes from session to session). However, the observation of consistent grip shape through static photographs does not preclude the possibility of high force variation, which is the case for participant 18 who only changed grip shape in the first three sessions, but exhibited high kinetic variability. Also, note that the intra-participant NCC values shown in Figure 5 are post-horizontal alignment; therefore, some participants such as participant 17, who rotated the pen as noted in Table 2, still surfaced as having a consistent grip shape (i.e., high intra-participant NCC values).

The plot on the right side of Figure 5 indicates the amount of variation between participants. Note that the overall inter-participant NCC values are lower than the intra-participant NCC values, indicating that the grip forces vary significantly between participants but are consistent within a participant. Some participants (6, 7, and 8) have very low inter-participant NCC values, suggesting that their kinetic grip shapes are very different from those of other participants.

**Figure 6:**
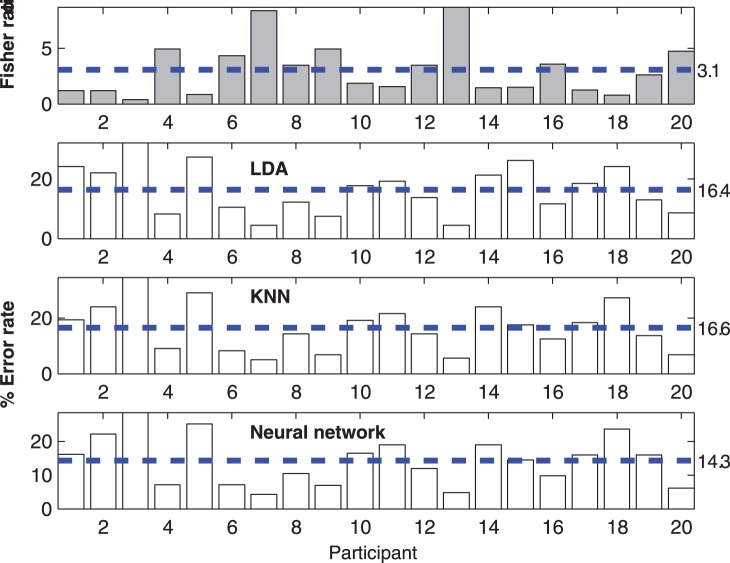
Separability of intra- and inter-participant NCC values. Separability measured by Fisher’s ratio (top graph) and classification error rates by LDA, KNN and NN classifiers (bottom three graphs respectively). The dashed line represents the average value of each method.

Figure 6 summarizes the Fisher’s ratio and the error rates associated with classifying NCC values for each of the 20 participants. The results from the different methods agree with each other; as expected, high Fisher’s ratio corresponds to low classification error rate. However, using the inter-image NCC value as an input feature generally leads to mediocre classification rates.

Table 3 summarizes the effect of grip shape image alignment on error rates associated with classifying the entire grip shape matrix. For all three classifiers, the error rates only decreased slightly after alignment, verifying our observations of circumferential offsets in grip shape but suggesting that these within-participant differences are not large enough to compromise image-based classification. Note however that the error rate for KNN grip shape matrix classification is much lower than that of the LDA and NN classifiers and generally much lower than that achievable with any classifier using NCC as input.

**Table 3:** Error rates in the classification of full grip shape images.

	LDA	KNN	NN
**Without grip** **shape alignment**	24.2±1.2	1.3±0.3	13.6±3.0
**With grip shape** **alignment**	22.2±1.3	1.2±0.4	12.9±1.1

Mean and standard deviation of error rates using LDA (linear discriminant analysis), KNN (K nearest neighbors) and NN (neural networks).

Particularly noteworthy is the fact that the intra-participant variability of some participants (1, 3, 6, 7, 8, 11, 17 and 18) decreased after horizontal alignment of grip shape images (slightly higher NCC values and fewer outliers). These were generally participants who were identified through photo review as having rotated the pen from session to session. Also, note that the handedness of the participant (right or left) did not have any particular effect on the grip shape variability.

## Discussion

In this paper, we studied the intra- and inter-participant variation in forces applied to the barrel of the pen during signature writing in adults, with a particular focus on the topographic distribution of forces. Kinetic data were collected on multiple days and at multiple times within-day.

### Grip Shapes

Nearly half the participants deployed a dynamic tripod grasp, while the remainder adopted quadrupod, lateral tripod or static tripod grasps. The predominance of grip shapes other than the dynamic tripod has also been found in a pediatric population [29]. In particular, 15% of participants adopted a lateral tripod grasp in our sample, which is on par with the fraction of lateral tripod writers that Bergmann [30] reported among 447 adults (without any known pathologies). The functional implications of grip shape on handwriting in adults is not well-documented at this time [31]. However, Stevens [32] did suggest that adults with a lateral tripod grasp may fatigue more quickly than those who employ other grip shapes in extended-duration writing tasks.

### Within-participant Variation of Grip Kinetics

The grip shape topographic maps and NCC results support the hypothesis that each participant possesses unique grip shape kinetics that are repeatable within-participant over time. This finding agrees with Schmidt and Lee [33], who contend that the relative force produced by muscles is an invariant feature of motor programs associated with a unique pattern of activity. Signature writing can be considered an example of such a learned motor program [34], rationalizing the observed within-individual kinetic consistency.

Our finding also aligns with [35] which examined the variation of handwriting grip patterns with age, from childhood through to adulthood. They observed a decrease in the variation of pen-surface positioning and the number of grips that individuals use as they mature, and speculated that this emerging invariance may be due to increasing automaticity and efficiency of handwriting as a manual motor skill. However, it is important to note that in [35] only a video-based grip classification scheme was used which did not consider the biomechanics associated with different pencil grip shapes. Finally, studies of grip forces associated with golf swings have shown that each player deploys a repeatable grip force profile that is distinct from that of other players [36]–[38]. Our finding corroborates the general conclusion of these studies that a high level of intra-participant grip kinetic repeatability tends to accompany a well-learned manual motor activity.

Despite the general finding of personal consistency, there was a degree of intra-participant variation. Hooke et al. [2] and Shim et al. [7] contend that pencil grip shape is governed by a kinetically redundant system; different combinations of finger forces and torques can generate similar kinematic and spatiotemporal profiles. Indeed multiple muscle groups, including those that move the fingers, wrist and forearm, are involved in the generation of hand kinetics and thus, kinetic variation invariably exists within individuals [39]. In [40], it was found that activation of both the extrinsic and intrinsic muscles of the hand is modulated by wrist angle during a two digit grasp. Thus, even in dynamic grasps where pen motion comes largely from finger articulation, forces may vary depending on the current wrist angle. Studies have also shown that for the control of multi-joint movements such as handwriting, proprioception plays a critical role [41], [42] and thus afferent inputs from the hand may also lead to variations in kinetic output.

Two participants (Figure 5) exhibited an inflated level of within-individual kinetic variability. Participant 6 had the lowest within-individual NCC. This can be explained by the participant’s tendency to modify his grip height and to alternate between grip shapes, specifically, extending or curling the index finger around the utensil. The participant confessed that these were habitual strategies to compensate for fatigue. Likewise, Participant 15 exhibited the widest variation of within-individual NCC. As noted in Table 2, this participant oscillated between various grip heights while writing. This observation also explains why horizontal alignment of the grip shape matrix did not reduce the within-individual NCC variation. The lack of familiarity with the instrumented pen, nervousness, or fatigue may have contributed to the use of multiple grip shapes while writing. Summers and Catarro [43] found that 28% of university students in their study used more than one grip shape during a 2 hour exam.

### Between-participant Variation of Grip Kinetics

Four different types of grip shapes were identified through retrospective picture review. However, an examination of the topographic distribution of the forces associated with each grip shape indicated that the mean grip shape images were distinct between participants even if two participants invoked the same grip shape. This kinetic uniqueness may be attributed in part to the personalized coordination of muscles in executing a complicated but well-trained motor skill such as handwriting and specifically signature writing [44]. With a three digit grasp, [45] posit that the distribution of neural drive to multiple hand muscles may reflect anatomical or functional properties of hand muscle groups, characteristics that are likely to vary among individuals and thus further contribute to unique grip shape images.

It is worthy to mention that Participants 6, 7 and 8 had the lowest inter-participant NCC, implying that their grip shape images were most unique among participants. Indeed, Participant 6 adopted a unique combination of a quadrupod grasp and minor variations thereof. Participant 7 preferred a tripod grasp but tended to hold the pen distal to the apex (Figure 4). Participant 8 on the other hand, held the pen nearly perpendicular to the tablet surface. These personal grip idiosyncrasies contributed to the distinct grip shape images of these three participants.

Using the full grip shape matrix as the input to a KNN classifier yielded much more compelling error rates (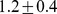 after horizontal alignment). This finding suggests that the boundaries among the individual grip shapes in 32-dimensional kinetic space are nonlinear. The low error rate also indicates that the entire force distribution provides a much more discriminatory feature set than that of a summary statistic (e.g., NCC). Indeed, handwriting requires multi-digit synergies [1], [46] that involve both intrinsic and extrinsic hand muscles [40]. The full grip shape matrix likely captures some of these interdigit coordination patterns that are missed by a simple summary statistic.

Our inter-participant variability findings echo earlier indications of significant between-participant variation in finger pressures during handwriting on the account of the personalized nature of this activity [47]. Further, Latash et al. [46] remarked that natural handwriting depends critically on the stability of individual-specific multi-digit synergies in which the fingers work as dependent force generators to stabilize the pen. Hence, kinetic variation across individuals is not unexpected. Such variation has also been noted with other motor skills such as golf swings [36]–[38].

### Possible Applications

The topographic representation and analysis presented herein may lead to new applications of handwriting grip kinetics in rehabilitation, biometrics and ergonomics. Building on the finding that certain handwriting disorders such as writers’ cramp are associated with abnormal finger postures and highly individualized grip shapes [8], topographical kinetic analyses that evaluate the subject’s grip shape and the extent of its variability may add to the clinical characterization of these conditions. Also a recent literature review on adults’ handwriting [31] pointed out the lack of handwriting research on healthy adults and the need for normative data. On these fronts, the present study contributes to the definition of typical variation in grip kinetics in adults, in the absence of handwriting pathologies. The uniqueness of grip kinetics may further inform the development of personalized fine motor interventions. For example, the prescription of different writing utensil adaptations (e.g., rubber or foam grips, indented pencils, triangular pencils, ring clips) may depend on the individual grip shape of the client. Kinetic topographies may also bear biometric value given that three dimensional forces of the pen tip have demonstrated potential for signature verification [48] and that pen tip and grip forces are strongly correlated [1]. Kinetic grip topographies, particularly, interdigit force synergies [49] may also inform hand grip designs that maximize comfort and performance for different hand sizes.

### Conclusion

In this paper, we introduced a topographic representation and image-based analysis of grip kinetics associated with adult signature writing. We conclude that despite day-to-day force variations within-individual, asymptomatic adult writers tend to exhibit a unique kinetic grip shape when writing. Further, these individual-specific kinetic grip shapes are algorithmically discernible from one another when the entire force distribution around the pen barrel is considered. The topographic analysis of grip kinetics may inform the development of personalized neuromotor interventions or customizable grips in clinical and industrial applications, respectively.
